# Innovative Visualizations Shed Light on Avian Nocturnal Migration

**DOI:** 10.1371/journal.pone.0160106

**Published:** 2016-08-24

**Authors:** Judy Shamoun-Baranes, Andrew Farnsworth, Bart Aelterman, Jose A. Alves, Kevin Azijn, Garrett Bernstein, Sérgio Branco, Peter Desmet, Adriaan M. Dokter, Kyle Horton, Steve Kelling, Jeffrey F. Kelly, Hidde Leijnse, Jingjing Rong, Daniel Sheldon, Wouter Van den Broeck, Jan Klaas Van Den Meersche, Benjamin Mark Van Doren, Hans van Gasteren

**Affiliations:** 1 Computational Geo-Ecology, Institute for Biodiversity and Ecosystem Dynamics, University of Amsterdam, Amsterdam, the Netherlands; 2 Information Science, Cornell Lab of Ornithology, Ithaca, New York, United States of America; 3 Information & Data Centre, Research Institute for Nature and Forest, Brussels, Belgium; 4 CESAM University of Aveiro, Aveiro, Portugal; 5 South Iceland Research Centre, University of Iceland, Selfoss, Iceland; 6 College of Information and Computer Sciences, University of Massachusetts, Amherst, Massachusetts, United States of America; 7 Electrical Engineering Department, University of Minho, Guimaraes, Portugal; 8 Oklahoma Biological Survey, University of Oklahoma, Norman, Oklahoma, United States of America; 9 Royal Netherlands Meteorological Institute, De Bilt, the Netherlands; 10 Mount Holyoke College, South Hadley, Massachusetts, United States of America; 11 iMinds, Ghent, Belgium; 12 Wildly Mild, Ninove, Belgium; Università della Tuscia, ITALY

## Abstract

Globally, billions of flying animals undergo seasonal migrations, many of which occur at night. The temporal and spatial scales at which migrations occur and our inability to directly observe these nocturnal movements makes monitoring and characterizing this critical period in migratory animals’ life cycles difficult. Remote sensing, therefore, has played an important role in our understanding of large-scale nocturnal bird migrations. Weather surveillance radar networks in Europe and North America have great potential for long-term low-cost monitoring of bird migration at scales that have previously been impossible to achieve. Such long-term monitoring, however, poses a number of challenges for the ornithological and ecological communities: how does one take advantage of this vast data resource, integrate information across multiple sensors and large spatial and temporal scales, and visually represent the data for interpretation and dissemination, considering the dynamic nature of migration? We assembled an interdisciplinary team of ecologists, meteorologists, computer scientists, and graphic designers to develop two different flow visualizations, which are interactive and open source, in order to create novel representations of broad-front nocturnal bird migration to address a primary impediment to long-term, large-scale nocturnal migration monitoring. We have applied these visualization techniques to mass bird migration events recorded by two different weather surveillance radar networks covering regions in Europe and North America. These applications show the flexibility and portability of such an approach. The visualizations provide an intuitive representation of the scale and dynamics of these complex systems, are easily accessible for a broad interest group, and are biologically insightful. Additionally, they facilitate fundamental ecological research, conservation, mitigation of human–wildlife conflicts, improvement of meteorological products, and public outreach, education, and engagement.

## Introduction

Migration is a salient feature of the life histories of many organisms, influencing biodiversity, ecosystem dynamics and functioning [1]. Monitoring the movements of these organisms has biological and applied value, such as understanding an organism’s capacity to adapt its behavior to internal and external factors during lengthy and stressful periods of movement, examining the effects of changing climates on behaviors of mobile organisms, or reducing conflicts between human activities and wildlife. Avian migration occurs on a global scale, with individual migratory routes that can span hemispheres, and its occurrence has been considered and documented in recorded history for centuries. However, most avian migration, as well as that of bats [2] and many aerial arthropods [3], occurs under the cover of darkness. The sheer magnitude of these movements and their occurrence at night, when most direct visual observation is impossible, poses major methodological and analytical challenges for quantifying and monitoring aerial migrations.

Radar (**ra**dio **detection a**nd **r**anging) is one of the few technologies available to monitor these incredible natural phenomena across appropriately diverse temporal and spatial scales and a broad range of species. Originally developed in the late 19^th^ and early 20^th^ centuries, radar was applied during World War II for surveillance of aircraft movements, and meteorologists and ecologists quickly discovered that the technology could be used to detect weather and birds aloft (e.g. [4, 5]). The radar emits pulses of radio waves that scatter against interfaces across which the dielectric constant changes, for example when reaching birds or other biota, precipitation or temperature inversions [6]. The position of targets in three-dimensional space is determined by their distance, which is estimated from the time it takes for the pulse to return to the antenna at the speed of light, together with knowledge of the antenna direction (elevation and azimuth). The amount of back-scattered energy can be used to estimate the amount of biological targets in a sampled volume. While medium and long-range radar are used to estimate the density of biological targets, they generally do not resolve individual targets. Velocity can also be extracted from radar data, although methods differ among radar systems. In summary, radar can provide accurate density and location information on biological targets in the air. For a more general background on radar terminology and the use of radar for ornithological research, see Bruderer 1997 [6].

Weather surveillance radar networks in Europe and North America provide near real-time information on the movements and distribution of precipitation and are used for weather monitoring and improving short to medium-range (e.g. 0–7 day) weather forecasts through assimilation in numerical weather prediction models. Operational weather surveillance radars also detect aerial biological targets [7–10]. When used in conjunction with data processing algorithms to extract biological information from these sensors, these networks can be used for long-term standardized monitoring of bird movement on a scale that has previously been unachievable [7, 11, 12]. Continental-scale networks for monitoring animal movement can likely be established at a relatively low cost, since the radar infrastructure is already in place and operational. Such networks will be invaluable for stakeholders interested in diverse applications, ranging from mitigation of potential hazards such as collisions with aircraft [13], conservation [14, 15] and fundamental science [16]. However, processing, visualizing and integrating the large volumes of data produced by such networks are still primary challenges. Analyzing a single night of migration across the continental US would require processing approximately 15,000 scans. The use of these large streams of remote sensing data for ecological research creates new opportunities and challenges for scientific discovery inherent to the era of big data, and will require appropriate infrastructure to store, organize, visualize, analyze, and share such immense datasets [17].

Visualizations can greatly facilitate data exploration, interpretation and scientific discovery, especially when datasets become large or multi-faceted. However, visualizing animal migration monitored with a radar network is challenging: a coherent picture of how animals move and how these movements change over time requires simultaneous interpretation of multiple variables (flight speed and direction, flight altitude, position, and time). Additionally, radars are unevenly distributed, have gaps in their coverage, or may provide only point estimates (e.g. vertical profiles of birds [9, 18]). Visualization techniques can help fill gaps in the data, integrate all information into a single display, and improve interpretability of the data. To achieve these goals, a visualization must be easy to use and reuse: it should be intuitive, interactive, easy to install or access in an online environment, applicable to diverse data sets, and open-source, so that it can be customized and adapted.

Radar technology has been used in a diverse range of studies of animal movement in the atmosphere that vary from local to continental scales. For example, radar studies have shown how the timing, flight speeds, orientation and altitudes of birds and arthropods are influenced by weather [19–22]. Radar combined with trajectory models have shown the large distances and ranges that can be reached by migrants within a few hours of flight [8, 23]. How birds react to landscape features such as rivers, lakes and coastlines and the identification of important stopover areas has also been revealed by radar studies [14–15, 24–26]. Increasingly, radar networks are used to study migration at broader spatial scales than single site studies and have revealed spatial differences in migration density as well as the flight speeds and orientation behavior of migrants [27].

Traditionally, ecologists have developed their own approaches for visualizing data from individual or several radars to portray information about animal migration in the air, such as timing, flight speeds, flight altitudes and density (e.g. [10, 19, 28–29]). For example, rose plots have been used to represent an integration of flight speeds (ground speed or air speed) and directions, providing a circular distribution of the movement vector (e.g. Figure 1 in [20]; Figure 4 in [21]; Figure 3 in [24]; Figure 5 in [27]), whereas histograms, boxplots or scatterplots have more often represented flight speeds or directions separately (e.g. Figure 6 in [23]; Figures 4, 5, 8 in [25]; Figures 3, 6, 8 in [22]; Figure 2 in [30]). Horizontal and vertical density have been represented in a variety of ways including histograms, bar charts, line plots and scatter plots (e.g. Figure 8 in [31]; Figure 2b in [10]; Figure 2a in [32]; Figure 2 in [33]). To provide more spatial information, density (horizontal or vertical) data have also been represented as raster (i.e. pixel grids in image files) and heat maps (e.g. Figure 3a,b in [9]; Figures 4–8 in [26]; Figure 3 in [29]; Figure 4 in [32]).

These data visualization formats are particularly useful for representing certain dimensions of the data, clearly exposing specific trends or other relevant patterns in migration. They are often limited however in their ability to represent multi-dimensional information and to integrate different aspects of the data, especially the dynamic aspect of migration in space and time. The aim of the current study was to develop novel visualization formats for migration research that represent multiple dimensions of the data in one comprehensive display, allowing the researcher to discern patterns which may have been previously impossible or much more difficult to identify. To this end, we assembled a multi-disciplinary team that developed two types of flow visualizations for a case study of mass nocturnal migration in Europe and then applied this approach to a case study of nocturnal migration in the United States. The data from the case studies are freely available and the visualizations are open source and thus can be explored and modified by others. Finally, we discuss the relevance of such visualizations for a diverse range of stakeholders and initiatives such as ENRAM (the European Network for Radar Surveillance of Animal Movement) [11] and BirdCast [27] that aim at establishing continental scale monitoring of aerial migration.

## Methods

In this section, we describe the processing of the radar data that was used as input for the visualizations of case studies of nocturnal bird migration from Europe and the US. We then describe two visualization methods that were developed for these case studies.

### European case study

For this case study we selected an example of nocturnal migration of birds over the Netherlands and Belgium in spring (see Figure 2 in [11]). We processed data from five operational weather radars (two in the Netherlands, three in Belgium) from 5 April 2013 00:00 UTC to 12 April 2013 00:00 UTC. We applied the methods described in [9] to process the radar data and extract altitude profiles of bird migration, assuming a radar cross section of 11 cm^2^ to convert reflectivity into bird densities (birds km^-3^), as established in a cross-calibration campaign for passerine migration [9]. We obtained estimates of bird density (*ρ*_bird_, birds km^-3^) and the horizontal ground speed components of flight (*u* and *v* components of bird velocity towards east and north respectively in m s^-1^) for every 5-minute period. We used all radar pulse volumes within 5–25 km of each radar station and for each 200-m altitude bin up to a sampling altitude of 4000 m above the surface using the methodology described in [9].

For the visualizations, we excluded the lowest altitude bin (0–200 m above surface level) to reduce the influence of ground clutter (radar reflectivity generated by objects on the ground) and included altitude bins up to 4000 m following [9, 34, 35]. When migration densities are low, speed measurements are more susceptible to contamination by residual rain or insects [9, 35]. Thus we retained *u* and *v* for *ρ*_bird_ ≥ 1 bird km^-3^.

After data processing and filtering, we aggregated data into 20-minute intervals and different altitude bands for each visualization (see description of visualizations below for more details about altitude bands). Within each altitudinal and 20-minute aggregation, we calculated mean *u* and *v*. If none of the altitude bins met the filtering criteria for speed as described above but did have a measurement of speed, we set the aggregated *u* and *v* to 0.

All data and metadata for this case study are deposited on GitHub (https://github.com/enram/case-study) and Zenodo (http://doi.org/10.5281/zenodo.57265), where it is released under a Creative Commons Zero waiver. The repository also includes code to process the data and a basemap for visualizations.

### United States case study

For this case study we selected an example of nocturnal autumn migration over the northeastern United States. We processed data from 13 Weather Surveillance Radar-1988 Doppler stations (hereafter WSR-88D) in the northeastern United States from the National Climatic Data Center from 8 September 2010 22:00 UTC to 11 September 02:00 UTC. We applied methods described in detail in [27] for downloading, screening, processing, and analyzing data for the US case study for all pulse volumes within a ~2–37.5 km radius from each radar station and up to 3000 m above the radar station. While these methods differed in some ways from those of [9], we produced analogous estimates of bird density (*ρ*_bird_) and *u* and *v* bird velocities for each 100 m altitude bin in each radar scan.

We further aggregated data for visualizations into different altitude bands (see description of visualizations for more details about altitude bands), by averaging bird density and *u* and *v* velocity over all altitude bins within the same altitude band and all scans within the same time interval (scan times are rounded to the nearest 5-minute interval, e.g. 9:10, 9:15, 9:20; it is possible but rare that two scans occurred within one such interval). In these averages we included *u* and *v* velocities only when *ρ*_bird_ ≥ 1 bird km^-3^, as in the European case study, to minimize the impact of residual rain and insects on the aggregate velocity measurements.

### Flow visualizations

Flow visualization techniques are commonly used to represent vector fields in various domains of science and engineering. To apply these techniques to visualize bird migration, one must address the issue that the radars only sample the value of the vector field at a small set of locations. Each sample is a point-vector pair with the point being the location of the radar and the vector being the migration velocity (the *u* and *v* components of ground speed) at a given altitude band. Given these sparse samples, the complete vector field can be reconstructed by means of interpolation. The migration density data can similarly be interpreted as a sparse scattered scalar field on the basis of which a complete scalar field can be reconstructed using interpolation. Both the velocity vector field and the density scalar field are time dependent for each altitude band.

We developed two visualizations that use local flow visualization techniques based on particle tracing [36]. The first visualization (‘Bird migration flow visualization’) shows a large number of animated streamlets, short streamline segments whose length is typically proportional to the flow magnitude (migration ground speed) at their seeding (i.e. origin) points. Streamlines are the paths traced by a particle moving through space and time according to the velocities given by the time-varying vector field. At any instant in time, the velocity vector at the particle’s current location is tangent to the streamline at that point.

The animated visualization incrementally moves this point through time to achieve a visualization of the dynamic vector field. The second visualization (‘Time Integrated Multi-Altitude Migration Patterns—TIMAMP’) shows a limited number of pathlines instead of streamlines. Unlike streamlines, which show moving particles and their recent tracks, pathlines record the movement tracks of particles over a longer period of time and display those paths on a fixed map. This allows changes in both the spatial and temporal dimensions to be shown in a single static visualization.

### Bird migration flow visualization

This visualization is a web application written in HTML, CSS and JavaScript, showing bird migration as an animated flow, superimposed on a map and progressing through time. Controls at the top of the visualization allow the user to start and stop this progression, manually navigate through time intervals, select a specific date and time, as well as toggle between two altitude bands. The code of the visualization is based on ‘air’, an open source project developed by Cameron Beccario to visualize wind and air pollutants in Tokyo (http://air.nullschool.net). The migration flow visualization has been adapted extensively from ‘air’ to support bird migration data, implement the user controls and especially progression through time.

The application requires three data files to visualize a case study: *basemap*.*topojson*, *radars*.*json*, and *birds*.*csv*. The file *basemap*.*topojson* contains the geographical features (such as country or state borders) to be displayed on a basemap for the visualization, while *radars*.*json* contains the position and identification for the radars that contribute to the case study. The geospatial data from both files are rendered using the Geo Projections API of the D3.js library (https://github.com/mbostock/d3/wiki/Geo-Projections) and are styled with CSS. The file *birds*.*csv* contains the filtered and aggregated bird migration data for each radar (indicated with *radar_id*) and is further aggregated in two altitude bands: (1) ≥ 0.2 km and < 1.6 km, and (2) ≥ 1.6 km.

**Fig 1:**
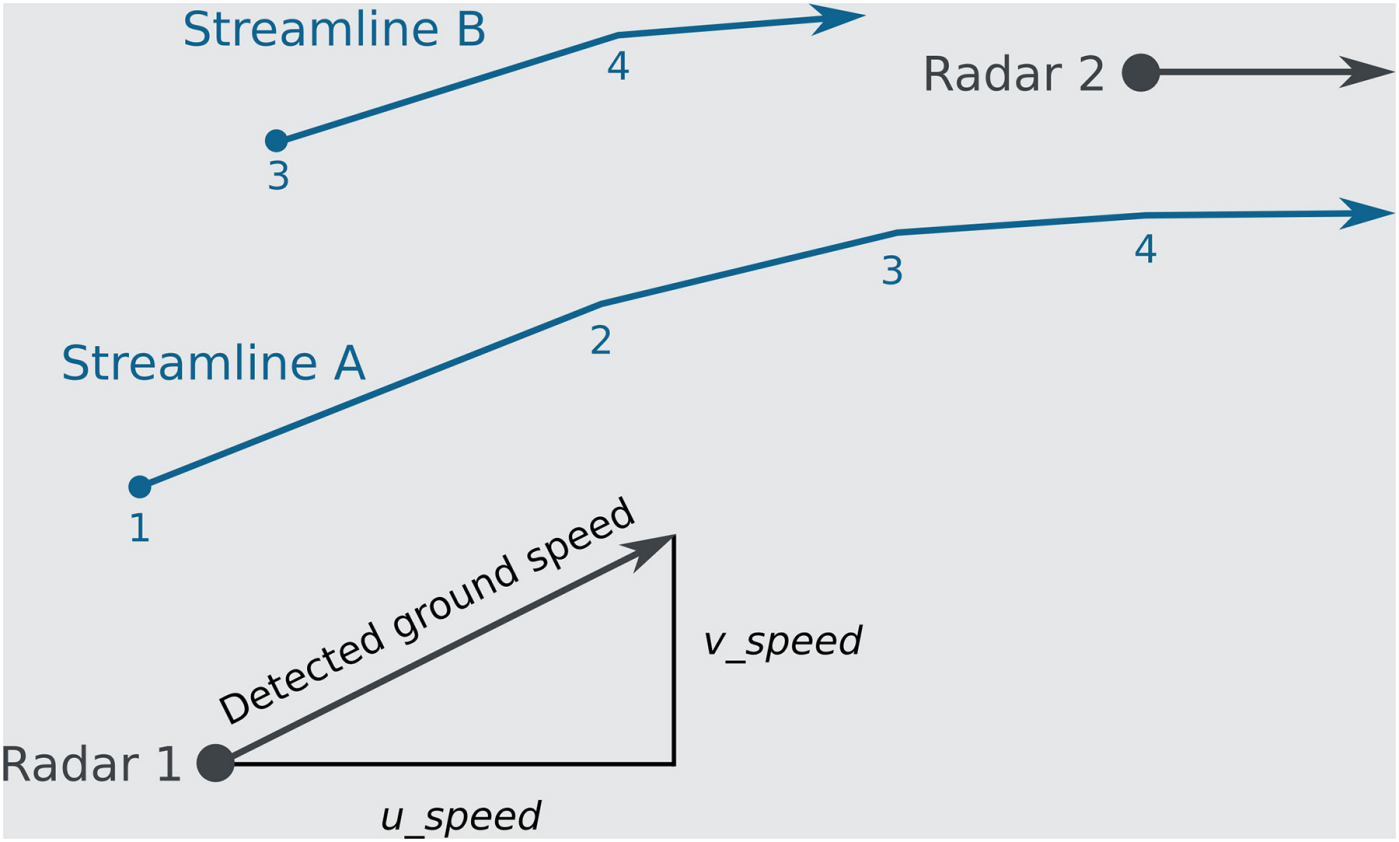
Schematic representation of the bird migration flow visualization. For each radar (Radar 1 & 2), the application will calculate a two-dimensional vector representing the *u* and *v* components of the detected ground speed of migration for that radar, altitude band and time interval (T0). Using the x and y positions of the radars, these vectors are interpolated using inverse squared distance weighting to create a vector field of interpolated ground speeds, expressed in pixels. Once the vector field is calculated, the animation starts. At animation frame 1, a streamline A (blue line) is initiated at a random position (blue dot) and the interpolated ground speed at that position is used to draw a line to a new position. This is repeated for each animation frame (2, 3, 4), creating a streamline through the vector field. At animation frame 3 an additional streamline B is initiated. After a fixed number of frames, the application will retrieve data from a new time interval (T1) and update the vector field, influencing the direction and speed of the streamlines.

Given an altitude band and time interval, the application will calculate a two-dimensional vector for each radar based on the *u* and *v* components of the detected ground speed of migration at that time (Fig 1). The x (*u* component) and y values (*v* component) of this vector are expressed in pixels and are independent from the scale of the basemap. Using the x and y positions of the radars, these vectors are then interpolated over a fixed rectangular grid using inverse (squared) distance weighting [37] to create a vector field. It is important to note that for every point in this grid, the resulting vector is calculated based on the data of the five closest radars. Note that radars with *u = 0* and *v* = 0 do affect the interpolation; in these cases measurements were taken, but no migration was detected.

**Fig 2:**
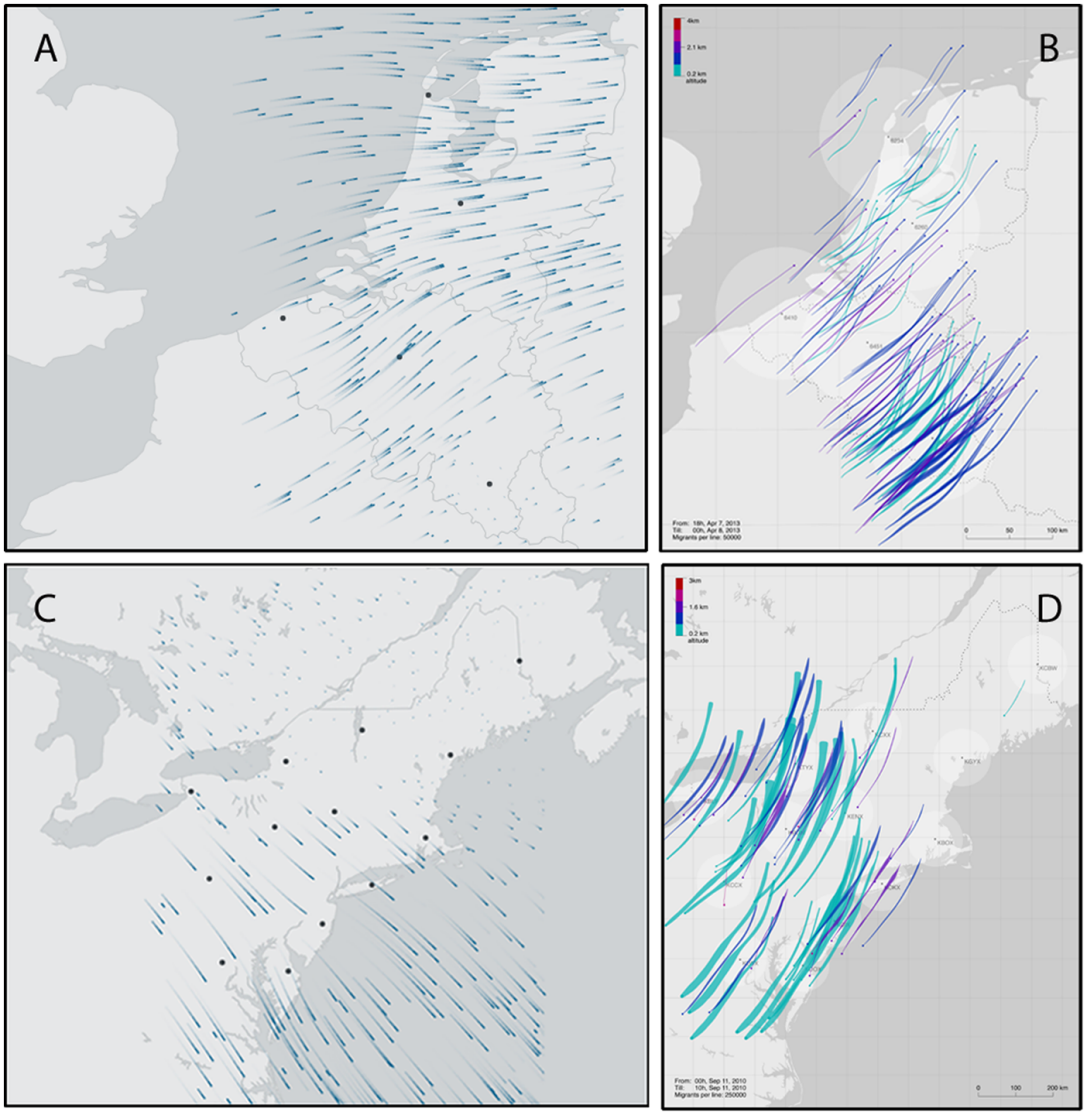
Nocturnal migration flow visualizations for Europe and the US. In all visualizations a black filled circle indicates radar location. (A) Bird migration flow visualization across Belgium and the Netherlands on 2013-04-07T05:00Z. The vector length is proportional to ground speed. Two different axes of nocturnal migration can be seen with birds arriving from the United Kingdom and travelling in an easterly direction and birds travelling along the main northeastern axis of migration. The northeastern axis can be seen in western Belgium and the west to east migration axis can be seen in the northern half of the Netherlands. Due to changes in mean ground speed and direction detected by the different radar the two flow patterns seem to converge. Note that in SE Belgium migration is almost absent. (B) TIMAMP visualization showing 6-hour migration flows in 5 strata (altitude bands) for Belgium and the Netherlands starting at 2013-04-07T18:00Z. Length and direction of lines represents the *u* and *v* components of ground speed integrated over 6 hours, the distribution of pathlines corresponds with relative density. Intense nocturnal migration occurs predominantly in SE Belgium across all altitude layers, with birds travelling at relatively high speeds. Migration track direction, however, differs between the lowest and higher altitude bands, with birds maintaining the primary northeast axis of migration at high altitudes but at the lowest altitude band in more northern areas and later in the night directions are shifted towards the north. (C) Bird migration flow visualization of the US case study showing nocturnal migration on 2010-09-09T03:45Z. Migration towards the southeast suggests initiation of over water migration, particularly from Cape Cod, Massachusetts to Chesapeake Bay, Virginia. Note that areas in northern New England, northern New York state, and the Lake Ontario region show substantially less migration activity than what occurred at more coastal locations. (D) TIMAMP visualization showing 10-hr migration flows in 5 strata for the US case study on 2010-09-11T00:00Z. Each pathline represents ca. 250,000 migrants. Migration directions during this period are typical of those described in Autumn for this region, with most birds moving toward south-southwesterly to southwesterly directions over land. Note, however, subtle change in migration direction across sites and during the night, as tracks arc more westerly as well as change in density during the night and across the region.

Once the first vector field is calculated, the application will randomly select 450 points in the field. For each following frame (with a desired frame rate of 60 m s^-1^), the application will: 1) use each point’s vector values to draw a line to its new position (Fig 1), 2) remove points that have reached a certain age, 3) create a new point in the field for each one that was removed, and 4) decrease the opacity of all previous lines. Combined, this creates an animated, steady flow of growing lines with varying lifespans whose trails fade as the animation progresses. The visualization progresses through time, recalculating the vector field for each time interval. Lines are created and fade away independently from this calculation, but the perceived flow evolves over time as the vector field updates regularly, corresponding to the measured changes in animal movement. For aesthetic purposes, the visualization flow speed is faster than measured speeds of animal movement and information on density is not used in this visualization.

All code and documentation for this visualization is deposited on GitHub (https://github.com/enram/bird-migration-flow-visualization) and Zenodo (DOI http://doi.org/10.5281/zenodo.57472), where it is licensed under an open source MIT license. The repository also includes processed data for the two case study. The version described in this paper is 2.0.

### Time Integrated Multi-Altitude Migration Patterns—TIMAMP

This static visualization is meant to provide an integrative picture of the spatial and temporal variation in migration activity during a period of one to eight hours. The visualization is implemented as an interactive web application written in HTML, CSS and JavaScript. It loads the same data as the ‘Bird migration flow visualization’. TIMAMP shows a number of pathlines on a geographic map. Each pathline represents the expected travel path of a virtual group of migrants during the selected time period. The user can interactively select the starting date and time, and the duration (in hours) of the visualized time period as well as the number of migrants represented by each pathline. The conversion from radar reflectivity to the number of migrants depends on the bird detection algorithm deployed and assumptions made regarding the type of migrants passing through the area see e.g. [9]; for the two case studies presented in this paper, the assumption is that migrants are predominantly passerines. The user can also select the number of altitude bands into which the full altitude range (0–4000 m) is divided. For each altitude band, a separate set of pathlines is drawn with a different colour, using the mean bird density and velocity data within that band. This function enables the user to conceptualize the variation among migration patterns at different altitudes.

We used the 2-stage Runge–Kutta algorithm to obtain the trajectory of a pathline, also known as the Heun method [38]. We used 20-minute time increments for this integration, matching the 20-minute time windows used for the temporal aggregation of the migration data. The integration proceeds backwards from an anchor point for the first half of these intervals, and forwards from that anchor point for the second half, yielding centrally anchored pathlines around each time step. The velocities used in the integration were interpolated from the sparse irregularly spaced data using inverse (squared) distance weighting, assuming a smooth continuous variation of the conditions that affect the migrant velocities (*u*, *v*) in between these samples, while considering that the sampled values hold for a considerable radius around the radars.

The visualization draws the pathline with a thickness that varies according to bird density during the selected time period. The density values were also interpolated using inverse distance weighting. The smooth curvature of the resulting pathline was obtained by applying cardinal spline interpolation [39]. A dot marks the endpoint of each pathline to indicate the direction towards which birds are moving. To determine the position of the pathlines, we segmented the geographic space in a lattice of rectangular areas (10x10 km for the European case study, 20x20 km for the United States case study). From these we only considered those that lie (mostly) within a 75 km radius around one of the radars. By combining this with the segmentation in altitude bands, we obtained a three dimensional grid of rectangular volumes. For each of these volumes, we interpolated the average bird density during the selected time period. By multiplying this number with the actual volume, we obtained an estimate of the average number of migrants observed in this volume during the selected time period. For each volume, we divided this quantity by the amount of migrants each path represents to obtain the probability with which a path is anchored in this volume. As such, the total number of migrants represented by the paths shown in a visualization approximates the average number of migrants observed during the selected time period in the vicinity of the radars, while their spatial distribution approximates the observed distribution.

## Results: Visualization Implementation

The best way to review the results is to explore the ‘Bird migration flow visualization’ and TIMAMP on the Internet, where they can be used interactively with a web browser and without the need to install additional software (Table 1). Furthermore, the web interface enables users to post comments or questions related to the Bird migration flow visualization. We provide an example below to show how these visualizations can be used to explore the spatio-temporal dynamics of bird migration (Fig 2). In both visualizations, the user can select the start time and altitude band of interest. In TIMAMP, the user can additionally control the duration of the data integration, the number of altitude bands in which the available data is organized, and the number of migrants each pathline represents (Table 1).

**Table 1:** Summary of migration flow visualizations. Information on user control functionality and terminology used in web interface in parentheses. Hyperlinks to online visualizations are provided.

User information	Bird migration flow visualization	TIMAMP
Selection of data and time Selection of altitude band Play and pause animation Integration time frame (h) Migrants/Path Comments section	Yes Yes (200–1600 m, > 1600m) Yes Not applicable Not applicable Yes	Yes Yes (number of strata, 6 options) Not applicable Yes (‘Window’) Yes (10, 25, 50, 100, 250, 500 x10^3^) No
European case study hyperlink	http://enram.github.io/bird-migration-flow-visualization/viz/2/nl-be/index.html	http://timamp.github.io/timamp/v2/case-eu.html
US case study hyperlink	http://enram.github.io/bird-migration-flow-visualization/viz/2/ne-us/index.html	http://timamp.github.io/timamp/v2/case-us.html

### European case study

During spring migration, the primary direction of nocturnal migration in the region is towards the northeast (approximately 41° from north), with occasional strong influxes of migrants from the United Kingdom travelling eastward [9, 34]. The primary axis of nocturnal migration is clearly visible in both visualizations across all five radars; at times, as described below, eastward migration across the North Sea from the UK can be seen across the Netherlands, but not Belgium. The time series begins on 5 April 2013 during a period with little or no nocturnal migration. The following night, 6 April 2013, begins with moderate migration towards the northeast along the primary axis of migration. Just after midnight, the bird migration flow visualization clearly shows eastward movement, which dominates the visualization in the Netherlands, whereas in central Belgium the northeast migration is still visible (Fig 2A, S1 Movie). These two flows are due to a mass arrival of birds from the UK, moving eastward in parts of the region, as well as a continuation of migration toward the northeast. Exploration of the data using the TIMAMP visualization shows that nocturnal migration density is highest at the inland sites in Belgium and the Netherlands; this is very clear on the night of 7–8 April (Fig 2B). Both visualizations emphasize that birds are travelling quite fast on this night, shown by the vector length. On this night, a migration layer develops at several radar sites, in which migration density is higher at upper altitudes than lower altitudes. Such layering events have been previously noted, predominantly in spring at single radar sites in temperate latitudes, and can be ascribed to specific weather conditions that result in opposing winds at the surface and more supporting winds aloft [19, 33]. An additional pattern is revealed in TIMAMP when exploring the 5-strata visualization on this night: flight directions at almost all sites, especially the inland site in southeastern Belgium, change with altitude (Fig 2B). This shows how complex migratory behavior can be even at this large spatial and temporal scale. The visualizations also highlight very odd patterns with contrasting migration flows (sometimes in opposite directions) within the region (see e.g. the bird migration flow visualization at 2013-04-06T17:40Z). Such patterns are difficult to explain in the context of broad-scale migration and may reflect local movements.

### United States case study

The two flow visualizations (Table 1) represent the time series of nocturnal bird migration in the northeastern US for four days and nights, spanning 8–11 September 2010. The bird migration flow visualization clearly shows that migration was primarily distributed over areas near the coast (i.e. over land and water) in the altitude band of 200–1600 m above the ground on 8–9 (Fig 2C) and 9–10 September. Whereas the primary axis of migration in this region is SSW in autumn [25], the bird migration flow visualization (Fig 2C) and TIMAMP show movements at several radars primarily directed offshore toward the southeast on 8–9 September. On this night, TIMAMP reveals a clear shift in flight direction during the night, from mostly southeasterly early in the night (until 2010-09-09T 04:00Z) to southerly and south-southwesterly later on. These movements suggest a transition from seaward departure in the first half of the night by species migrating south over the western North Atlantic to primarily overland migration during the second half of the night (e.g. [40–42]). Given that the predominant flight direction in this region is southwesterly, and that it changes over the course of the night, conventional static figures generated at seasonal or even daily resolution are likely to miss these light, early-night southeasterly movements.

On 10–11 September, the bird migration flow visualization as well as TIMAMP reveal fast southwesterly movement across all stations, exhibiting a typical pattern for flight directions of migration across the region (see details in [27]). This contrasts dramatically with the flight directions of the previous nights’ movements, as does the migration density, which is much higher and clearly shown in TIMAMP (Fig 2D). Additionally, the bird migration flow visualization shows that the speeds of migration in this night’s movements are noticeably faster than on previous nights and that movement is distributed across the entirety of the region. Furthermore, the ease of incrementing through time on the TIMAMP visualization again reveals that the overall direction of migration changes clockwise over the course of the night, from south-southwesterly to southwesterly; this insight is not as easily gained from conventional visualization methods. Both visualizations reveal subtle differences in flight direction in Massachusetts compared to other areas from 2010-09-10T04:00Z to 2010-09-10T07:00Z, which would be difficult to observe when exploring individual sites separately with static Figures. Exploring the 6h time integration of TIMAMP on the night of 10–11 September reveals how flight direction and migration density differ across the region as well as within an area during the night (Fig 2D).

## Discussion

Traditional approaches to highlight details of animal migration have been limited by their static nature, focusing often on one dimension of information at a time. Yet recent advances in data visualization have led to increasingly intuitive, accessible, and aesthetic representations of multidimensional data [36, 43], providing inspiration for the current work. The flow visualizations developed here highlight the dynamic aspects of animal movement in space and time in a way that has not been previously realized in more traditional and typical visualizations. The web-based tools that we developed enable a more realistic, multi-dimensional representations of data, allowing for easier interpretation and faster review across multiple radar stations (see e.g. the automated progression of the bird migration flow visualization through time, S1 Movie). The flow visualizations provide an intuitive way to explore these patterns, enriching the viewers’ experiences from multiple perspectives and facilitating the integration of information across multiple sensors in space and in time—a necessity for exploring and interpreting these large and complex datasets. TIMAMP, for example, enables the user to select different altitude aggregations and time intervals for data integration in the same visualization, showing dynamics in space and time in one static display. Due to the multi-dimensional representation of data, the visualizations can also be used to draw attention to unusual patterns in the data that may only reach the attention of a researcher when exploring multiple sites simultaneously, such as the flow patterns in opposing directions noted in the European case study.

In future developments of such flow visualizations there are several aspects that require attention, including, for example, an improved representation of the density of aerial organisms at different spatial and temporal scales without over-cluttering visualizations. Currently, density is not included in the bird migration flow visualization, which may result in occasional over interpretation of high-speed low migration densities that may disproportionately influence the visualization. Similarly, as weather and especially wind can have a strong impact on migratory movements [44–46], incorporating information on wind fields and other environmental conditions can improve data interpretation. The addition of new data layers, especially in an operational and open source setting, will also depend on data availability. Regardless, further developments and research are needed to find the right balance between information content and information transfer through intuitive visualizations.

Development of the tools to visualize the dynamic nature of bird migration resulted from intensive interdisciplinary collaboration. Diverse expertise from biological, computer science, and graphic design backgrounds was required to create biologically meaningful and esthetic visualizations. Although the challenges of combining diverse backgrounds of all partners may initially impede progress (i.e. the creation of tools), all collaborators can benefit from group initiatives [47] geared at different target audiences. For the current study, we organized our group activities as hackathons, allowing rapid code development and feedback between the software developers and researchers. We also used GitHub as a platform to openly discuss tasks and issues, as well as storing version code and documentation, facilitating outside contributions and references. The resulting visualizations were made available publicly and announced via social media for additional outreach and exposure, and disseminated at diverse conferences and workshops. These methods for collaboration and communication complement peer-reviewed publications: they often reach a broader audience than most peer-reviewed publication outlets alone and highlight the outcomes of work that might otherwise be overlooked or found only in the methods’ sections of scientific publications and conference proceedings (e.g. [48]).

The flow visualizations we developed may have immediate value and application for advancing aeroecology and conservation. As the classification of biological targets improves (e.g. distinguishing among birds, bats and insects), so too will the quality of the data presented in the visualization and the range of biological movement patterns that can be represented. These tools can be used to help researchers investigate data quality and identify potential issues related to data collection and processing in projects in progress, and not just for presenting final results. By visualizing data dynamically and simultaneously across multiple radar we expect that these visualizations will be invaluable for exploring and identifying the influence of synoptic scale weather patterns on migratory behavior [46]. To facilitate joint discovery and exploration, not only should data, code and visualizations be present but an online forum to discuss findings, questions and suggestions for improvements openly. Such an iterative approach to data exploration would facilitate aeroecological research [49] and improve the exchange of expertise in this highly interdisciplinary field.

Our study shows that these methods can be applied to new case studies on different continents if the appropriate data are available. Web based services and JavaScript make these visualizations portable, as they do not require installation of special software and can be viewed in a broad range of browsers and devices. Moreover, our method is scalable and transferrable and can accommodate longer time series, larger spatial extents, and larger sensor networks. These visualizations will strongly support the efforts of ENRAM [11] and of BirdCast [27], projects that aim to monitor, study and forecast continental scale aerial migration for a range of stakeholders. We believe that our visualization tools can be operationalized and integrated into a system that includes streaming of weather radar data products, data processing to extract biological information, data storage, and near real time visualizations, making an even greater connection between the visualization and the reality of these movements. For example, NOAA radar data are available freely for the continental US as they are produced and have been archived since 1991, making the step to an operational near real-time system feasible. In Europe data availability is still a topic of discussion and a systematic long-term archive of weather radar data is not yet available at the continental scale but is something the ENRAM community [11] is trying to achieve. We expect that advances in data sharing policies and parallel processing, delivered predominantly through cloud computing, will be instrumental in making such systems broadly accessible and their data more efficiently processed [50].

## Concluding Remarks

Migratory animals link distant ecosystems, affect community dynamics and ecosystem functioning, and often serve as bio-indicators of human impact on land use and climate, yet these interactions are complex and often understudied [1]. Creating an easy and intuitive means to access the dynamic nature of migratory movements has great potential value for a diverse array of stakeholders, including military and civil aviation [13, 51], the wind energy industry [52], and conservation practitioners [53]. Furthermore, having tools that enable exploration of radar data archives and visualization of the massive quantity of information may provide new insights into how anthropogenic global change is impacting populations of migratory birds. Weather surveillance radars are already detecting broad front movement of birds, large-scale movements of agriculturally important insects, and movements of bats from hibernacula [8, 10, 29, 27]. With our tools one can depict the flow of animal movement at broad spatial and temporal scales, for use in scientific research, conservation and risk mitigation and for general inspiration. Visualizations produced by these tools can garner significant attention in the media and public at large, and can be used as a means to engage a discussion about topics like nocturnal migrations that may be otherwise difficult to convey and discuss meaningfully.

## Supporting Information

S1 Movie An animated gif of nocturnal migration in Europe using the bird migration flow visualization. The animation represents 12 hours from 2013-04-06 19:00 UTC to 2013-04-07 07:00 UTC.
(GIF)
