# Supplementary figures and images for: Innovative Visualizations Shed Light on Avian Nocturnal Migration
Source: PLoS One. 2016 Aug 24;11(8):e0160106. doi: 10.1371/journal.pone.0160106 (PMC4996449; doi:10.1371/journal.pone.0160106)

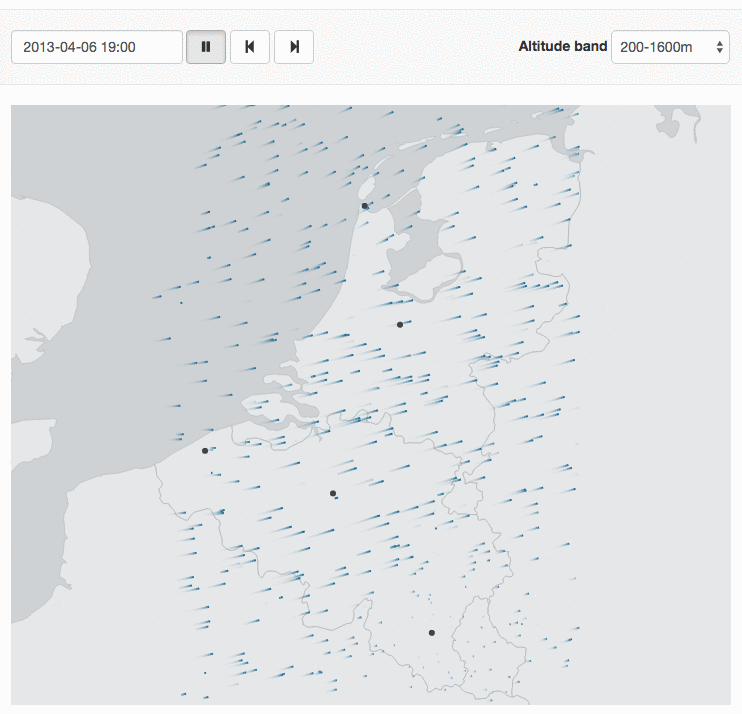

Supplement: S1 Movie — The animation represents 12 hours from 2013-04-06 19:00 UTC to 2013-04-07 07:00 UTC. (GIF) [file pone.0160106.s001.gif]
